# Transcriptomic Analyses during the Transition from Biomass Production to Lipid Accumulation in the Oleaginous Yeast *Yarrowia lipolytica*

**DOI:** 10.1371/journal.pone.0027966

**Published:** 2011-11-22

**Authors:** Nicolas Morin, Julien Cescut, Athanasios Beopoulos, Gaëlle Lelandais, Veronique Le Berre, Jean-Louis Uribelarrea, Carole Molina-Jouve, Jean-Marc Nicaud

**Affiliations:** 1 INRA, UMR1319 Micalis, Jouy-en-Josas, France; 2 Université de Toulouse, INSA, UPS, INP, LISBP, Toulouse, France; 3 INRA, UMR792, Ingénierie des Systèmes Biologiques et des Procédés, Toulouse, France; 4 CNRS, UMR5504, Toulouse, France; 5 Dynamique des Structures et Interactions des Macromolécules Biologiques, UMR-S 665 - Université Paris 7, INTS, Paris, France; 6 Plateforme Biopuces de la Génopole de Toulouse Midi Pyrénées, INSA/DGBA 135, Toulouse, France; 7 CNRS, Micalis, Jouy-en-Josas, France; Texas A&M University, United States of America

## Abstract

We previously developed a fermentation protocol for lipid accumulation in the oleaginous yeast *Y. lipolytica*. This process was used to perform transcriptomic time-course analyses to explore gene expression in *Y. lipolytica* during the transition from biomass production to lipid accumulation. In this experiment, a biomass concentration of 54.6 g_CDW_/l, with 0.18 g/g_CDW_ lipid was obtained in *ca.* 32 h, with low citric acid production. A transcriptomic profiling was performed on 11 samples throughout the fermentation. Through statistical analyses, 569 genes were highlighted as differentially expressed at one point during the time course of the experiment. These genes were classified into 9 clusters, according to their expression profiles. The combination of macroscopic and transcriptomic profiles highlighted 4 major steps in the culture: (i) a growth phase, (ii) a transition phase, (iii) an early lipid accumulation phase, characterized by an increase in nitrogen metabolism, together with strong repression of protein production and activity; (iv) a late lipid accumulation phase, characterized by the rerouting of carbon fluxes within cells. This study explores the potential of *Y. lipolytica* as an alternative oil producer, by identifying, at the transcriptomic level, the genes potentially involved in the metabolism of oleaginous species.

## Introduction

In a context of increasing concern about global warming and dwindling stocks of fossil fuels, the search for alternative, renewable sources of energy is now a matter of the utmost importance for modern societies. Biodiesel has rapidly become one of the most promising and widely studied alternative sources of energy.

Commonly, biodiesel is produced from refined or edible oils using methanol and an alkaline catalyst. However, the large-scale production of biodiesel requires considerable amounts of these oils, resulting in a sharp increase of their demand over the last decade. The eventual goal is to gain access to a sustainable energy source, as traditional methods of obtaining oils from plants have raised unexpected ecological and sociological issues (*e.g.* extensive use of arable land, replacement of food crops with fat-producing crops for biofuel production). As a consequence, the development of new production procedures from non-edible oils appears to be an essential prerequisite for a sustainable biodiesel industry. To that end, several esterification processes are currently developed for the utilization of these oils, often containing free fatty acids (FFA) (*e.g.* two-step esterification process [1], supercritical methanol esterification [2], lipase esterification [3]).

Microorganisms, including yeasts in particular, have long been studied as alternative sources of oils and fats [4], [5]. Under specific conditions, they synthesize and store lipids in the form of triacylglycerols (TAG) and sterol esters (SE) inside a special compartment of the cell, called the lipid body (LB). These neutral lipids serve as energy source for the cell when required. Some species have been reported to accumulate more than 20% of their dry cell mass in the form of lipids, and have been classified as “oleaginous” microorganisms [6], [7]. Oleaginous yeast species (*e.g. Rhodotorula glutinis*, *Lipomyces starkeyi*) are particularly promising in this respect, as they can accumulate more than 70% of their dry cell weight as lipids [7]. Additionally, they can present various fatty acid profiles, depending on the species and/or growth conditions. These features, combined with the ease of genetic manipulation and cultivation, make yeasts a target of choice for potential applications like nutritional supplements production (*e.g.* production of polyunsaturated fatty acids), or as oil providers for biodiesel production [3], [4].

*Yarrowia lipolytica* is one of the most widely studied “nonconventional” oleaginous yeast species [8], [9]. It has been isolated from various food-related environments (*e.g.* cheese, sausage), but also from sewage, soils and oil fields [10]. Its classification by the American Food and Drug Administration as “Generally Recognized As Safe” (GRAS) paved the way for the development of various biotechnological applications, including (i) heterologous protein production [11], (ii) organic acids production [12], and (iii) single-cell oil productions from agroindustrial by-products or wastes [13]. Under specific growth conditions, *Y. lipolytica* accumulates large amounts of lipid, sometimes accounting for more than 50% of its dry cell weight [14]. One of the major advantages of this yeast is its ability to use hydrophobic substrates (*e.g.* alkanes, fatty acids, oils) efficiently as a sole carbon source [10], [15]. *Y. lipolytica* cells accumulate large amounts of lipids on these substrates, using specialized protrusions formed on their cell surface to facilitate the uptake of hydrophobic compounds [16]. These characteristics, together with the availability of the complete genome sequence through the work of the Genolevure Consortium [17], render *Y. lipolytica* a model of choice for investigations of lipid accumulation in oleaginous yeast species. Various studies have already made use of the genome sequence to decipher aspects of lipid metabolism in *Y. lipolytica*, and some of the genes involved in the bioconversion, synthesis and mobilization of lipids have been described [18]. Furthermore, the availability of genetic information makes this yeast a suitable candidate for genetic and metabolic engineering approaches aiming to develop optimized yeast strains for the production and storage of large amounts of lipid with a specific fatty acid (FA) composition [15]. In addition, lipid composition of *Y. lipolytica* is mainly consisted of the C16–C18 fatty acid families, making this yeast attractive to biotechnological applications, such as biodiesel production [19].

Oleaginous microorganisms have been studied over decades, but the general mechanisms underlying their metabolic specificities remain unclear. Lipid accumulation has been described as a consequence of the slower growth observed when oleaginous organisms are subject to nutrient shortages (*e.g.* nitrogen deficiency) while growing on an excess of carbohydrates [7]. It has therefore been suggested that lipid accumulation in oleaginous organisms is the consequence of a stress response or of an adaptation to a nutrient shift [20]. Only a few studies have focused on the proteomics of lipid accumulation in oleaginous yeasts [20], [21]. Some attempts have been made to describe potential set-ups for the large-scale production of single cell oils (SCO) [22]. However, the optimization of these set-ups will require improvements in our understanding of the phenomena involved at the various “omics” levels. In particular, a complete transcriptomic study appears essential to an understanding of the cascade of metabolic processes involved in the transition from growth to lipid accumulation, for insight into regulatory mechanisms and, ultimately, for the identification of targets of interests for further genetic and metabolic engineering.

In this study, a controlled fed-batch culture was carried out to monitor the transition from biomass production to lipid accumulation under defined conditions. Storage lipid and transcriptomic time-course analyses were performed to explore the accumulation and metabolism of lipids in *Y. lipolytica*. This study constitutes the first attempt to unravel the transcriptomic response of an oleaginous yeast during its metabolic shift from growth to lipid accumulation.

## Materials and Methods

### Strain, growth conditions, and fed-batch culture strategy

*Y. lipolytica* mutant strain JMY1346 was used in this study. JMY1346 is a prototroph derivative of strain JMY1202, which was previously obtained by deletion of the *GUT2* gene in the auxotrophic strain Po1d (Leu^−^, Ura^−^) [18]. One frozen stock culture was used for the inoculation of a two-step preculture, which was further used to inoculate the fed-batch in defined mineral medium (see Table S1) [23].

Fed-batch cultures were performed in a 20 l bioreactor, with the Braun Biostat E fermenting system (Braun, Germany), without oxygen limitation. The temperature was set to 28°C and the pH to 5.8. Custom-built software was used for online acquisition and regulation of the controlled parameters (*i.e.* stirring rate, pH, temperature, relative pressure, partial pressure of dissolved oxygen, additions of bases and antifoaming agent). Relative pressure in the bioreactor was maintained at 0.3 bar. No more than 0.2 ml of antifoaming agent (Struktol JG73, Schill+Seilacher group, Germany) was added to the culture. During fed-batch culture, the bioreactor was supplied with three sterile feeds (glucose, salt and base, *i.e.* ammonia or potassium hydroxide), *via* Masterflex and Gilson peristaltic pumps (Cole-Parmer Instrument Company, USA, Gilson Inc., USA). Glucose feed concentration was 740 g.l^−1^. The masses of glucose and nitrogen added to the fermentor were estimated online, by weighing (CPA16001S, Sartorius, Germany). Outlet gas composition (after condensation) was analyzed by mass spectrometry (PRIMA 600 s, VG Gas, United Kingdom). O_2_ consumption and CO_2_ production rates were calculated from mass balances, taking into account changes in gas volume in the reactor, inlet airflow (as measured with a mass flow meter, Brooks, USA), temperature, humidity and pressure. The glucose concentration within the bioreactor was evaluated with custom-built software based on carbon mass balance and taking into account various data acquired online (*i.e.* glucose mass, gas analysis and inlet/outlet gas flow).

The fed-batch culture was divided into three phases, based on different carbon and nitrogen feeding strategies [23]: (I) growth phase, (II) transition phase, and (III) nitrogen limitation. During the growth phase (I), glucose flow was exponential, to ensure a constant specific growth rate without nutrient limitation. Nitrogen was supplied *via* 10 mol.l^−1^ ammonia solution, which was also used for pH regulation. The transition phase (II) corresponded to nitrogen limitation in the presence of excess carbon. This phase could be divided into two parts: (i) a decrease in the nitrogen concentration of the broth, (ii) a transition of nitrogen input from the pH regulation pump to an independent peristaltic pump. The starting point of phase II may thus be considered to correspond to the beginning of nitrogen depletion, triggered by shifting the pH-regulating solution from ammonia (10 mol.l^−1^) to potassium hydroxide (10 mol.l^−1^). Once the nitrogen concentration fell below 10 mmol.l^−1^, a controlled supply of 5 mol.l^−1^ ammonia solution was initiated, leading to a stabilization of the Carbon/Nitrogen (C/N) ratio near *ca.* 30 Cmol.mol^−1^. The lipid accumulation phase (III) began when the C/N ratio reached *ca.* 20 Cmol.mol^−1^, as established by Granger *et al.* [24]. After 33 h, the glucose supply was limited, to stop lipid accumulation. Throughout the experiment, samples (*ca.* 300 mg of cell dry weight) were harvested, frozen in liquid nitrogen, and stored at −80°C.

### Biomass analyses

Biomass production was determined by measuring A_600_ and cell dry weight, as estimated for three replicates after filtration and drying (200 mmHg, 60°C, for 48 h, until a constant weight was reached). Ash composition was determined after two complete combustions in a muffle furnace at 550°C for 12 h, in the presence of 200 µl of a 20 g.l^−1^ NH_4_NO_3_ solution. The biomass formula was determined by elemental analysis of C, H, O and N. The total fatty acid content of the dried samples was analyzed as described previously [25].

### Supernatant analyses

The sugar and organic acid concentrations of filtered supernatants were determined by HPLC (Ultimate 3000, Dionex, USA) with an Aminex HPX-87H+ column (Bio-Rad, USA), under the following conditions: 50°C, with 5 mM H_2_SO_4_ as the eluent (flow rate 0.5 ml.min^−1^) and dual detection with a refractometer at 50°C (Shodex, Japan) and UV measurement at 210 nm (Dionex, USA). Standards were used for compound identification and quantification. The glucose concentration of culture supernatants was also determined with a YSI Model 27 A glucose analyzer (Yellow Springs Instruments, USA). The residual ammonium concentration in the culture medium was determined with an ammonium ion electrode (PH/ISE meter model 710A+Ammonia Gas-Sensing Electrode Model 95-12, Orion Research Inc., USA). A combination of the various macroscopic analyses was used to calculate carbon mass and redox balances with a maximal error of *ca.* 4%.

### RNA extractions and microarrays

We used 11 sampling points, regularly spaced over the period of fed-batch culture, for transcriptomic analysis. Frozen samples were mechanically disrupted with a bead beater (Microdismenbrator, Braun, Germany) and a tungsten bead (Ø∼7 mm), for 2 min, at 2600 rpm. RNA was extracted from the resulting powder with the RNEasy Midi Kit (Qiagen, The Netherlands). The quality and quantity of RNA were assessed by capillary microelectrophoresis, with an RNA 600 Nano LabChips and a Bioanalyzer 2100 (Agilent, USA). The mRNA obtained was reverse transcribed and labeled using the ChipShot™ Direct Labeling kit (Promega, USA). Each sample was labeled with Cy5, and a mixture of all the RNA samples was labeled with Cy3 and used as a reference. The resulting labelled cDNAs were further purified using the ChipShot™ membrane Clean-up system (Promega, USA). Microarray probes were designed and slides were produced by Eurogentec (Belgium). Samples were hybridized in the Discovery XT System (Ventana Medical Systems Inc., Roche, Switzerland). Microarray slides were prehybridized with a solution of 2× SSC, 0.2% SDS, 1% BSA at 42°C for 30 min. 200 µl of the hybridization solution, containing 20 µL of labeled cDNA (50 pmol of Cy3 reference sample and 50 pmol of Cy5 sample of interest) and 180 µL of hybridization buffer (Chyp Hyb buffer, Ventana Medical System) were added on the printed side of the slide. Arrays were scanned with an Innoscan 700 Microarray Scanner (Innopsys, France) and fluorescence was measured with GenePix Pro v3.0 software (Molecular Devices, USA).

### Data filtration, normalization and statistical analyses

Raw transcriptomic data were filtered and normalized with R software [26], and the Limma package of the Bioconductor library [27], [28]. A preliminary filtration of the dataset was carried out with the quality flags provided by GenePix software. Spots with a quality flag value below “0” were removed from the analysis. Local background estimates were corrected by the “normexp+offset” method, using an offset value of 50 [29]. Background levels were subtracted from the data, which were then further normalized by the PrintTip Loess method [30]. Where possible, missing values for the filtration and normalization processes were simulated with the iKNN algorithm [31]. The normalized data were analyzed further, with MeV software [32]. First, the samples were clustered hierarchically on the basis of mean linkage and Euclidean distance. A 100 dendrograms were simulated for the calculation of bootstrap values, and the resulting tree was drawn with the “ape” package for R [33]. Samples were group based on the resolved clusters, and two-class unpaired Significance Analysis of Microarrays (SAM) tests [34] were performed between the resulting groups of samples. Genes were identified as differentially expressed in cases of significant detection, with a false discovery rate lower than 1×10^−5^ and an absolute fold change between two groups of more than 1.5. K-means clustering was performed on the expression profiles of the identified genes, using Pearson correlation as distance metric, and a maximum number of 50 iterations as convergence criteria. Functional classification of the genes was performed according to Gene Ontology Terms [35] defined during the genome annotation of *Y. lipolytica* [17], and by comparisons with homologous genes in *S. cerevisiae*. The data discussed in this publication have been deposited in NCBI's Gene Expression Omnibus [36] and are accessible through GEO Series accession number GSE29046 (http://www.ncbi.nlm.nih.gov/geo/query/acc.cgi?acc=GSE29046).

## Results

### A fed-batch lipid accumulation process for transcriptomic analysis

In oleaginous microorganisms, the initiation of lipid accumulation during lipid synthesis is caused by the exhaustion of a primary nutrient from the culture medium. Although many nutrients can be limiting, nitrogen limitation is the easiest condition to control and is generally the most efficient type of limitation for inducing lipid accumulation. During the growth phase, the carbon flux is distributed between the four macromolecular pools (carbohydrate, lipid, nucleic acid, protein). When nitrogen becomes unavailable, the catalytic growth rate slows down rapidly, whereas the rate of carbon assimilation slows more gradually [7], [37]. This results in the preferential channelling of carbon flux toward lipid synthesis, leading to an accumulation of triacylglycerols within the lipid body of the cell. When carbon is present in large excess, its uptake is limited only by the substrate transport system of the cell. In this case, limiting concentrations of nitrogen in the medium lead to the induction of lipid accumulation. The critical nitrogen concentration for lipid induction in *Y. lipolytica* has been found to be about 10^−3^ mol l^−1^ [23]. It is important for nitrogen concentration to exceed this threshold value to prevent the production of secondary metabolites (citric acid) that will otherwise affect lipid accumulation.

During the transition between the growth phase (growth with the production of catalytic biomass) and the lipid accumulation phase (decrease in growth rate due to nutrient limitation and the diversion of excess carbon to lipid production), some pathways are repressed (nucleic acid and protein synthesis), whereas others are induced (fatty acid and triacylglycerol synthesis). When non-oleaginous microorganisms are placed in the same nutrient-limiting conditions the available carbohydrate substrate, is diverted into various polysaccharides, including glycogen and various glucans and mannans. Here, we try to identify, at the transcriptomic level, the genes potentially accountable of the oleaginous character of the cell in *Y. lipolytica*.

**Figure 1:**
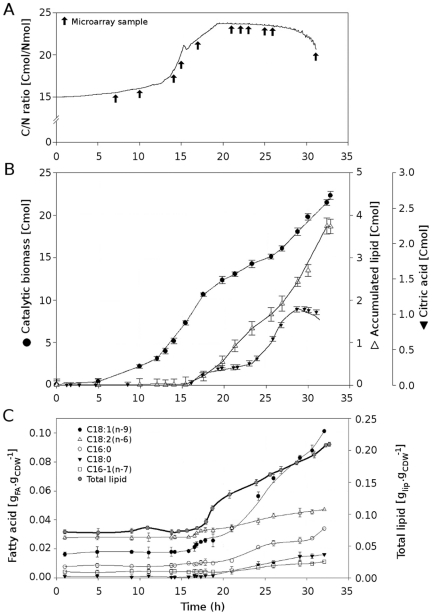
Macrokinetic and storage lipid analyses of the fed-batch culture. (A) changes in C/N ratio, as monitored during the fed-batch culture; (B) catalytic biomass, accumulated lipid and citric acid production; (C) total lipid and fatty acid production. Mean values and error bars were calculated using three replicates.

Growth and lipid accumulation were monitored during the culture (Figure 1). Evolution of parameters such as C/N ratio, biomass production and lipid accumulation can be divided into three major phases: (i) a growth phase, from the start of the culture to *ca.* 15 h, (ii) a short transition phase and (iii) a lipid accumulation phase from *ca.* 18 to 33 h. The first phase was characterized by an exponential increase of biomass, with a specific growth rate of 0.27 h^−1^, and a stable C/N consumption ratio of 15.5±1 Cmol/Nmol (Figure 1A and 1B). During this phase, lipid level and composition remained stable (Figure 1C). The second phase can be described as the entry into nitrogen limitation (15 h), resulting in a break in the biomass accumulation curve, with a decrease in specific growth rate from 0.27 h^−1^ to 0.07 h^−1^. The cessation of nitrogen feeding was effective from *ca.* 15 h (Figure 1A), but the nitrogen supply was not exhausted until 18.5 h. This second phase can be considered as an adaptation period, corresponding to the time required for the biomass to use all of the nitrogen present in the medium. During this phase, the C/N consumption ratio increased to 23 Cmol/Nmol (Figure 1A). The third phase began when lipid accumulation effectively increased, starting from 18.7 h (Figure 1B). Once the lipid production phase had begun, lipid content appeared to increase steadily until *ca.* 33 h. This pattern of lipid accumulation was linked to the linear glucose and nitrogen feed (*i.e.* constant C/N ratio, as described in Figure 1A). Maximum glucose concentration was below 0.2 g.l^−1^ during phase III, with the accumulation of less than 1 Cmol of citric acid (Figure 1B), *i.e.* 2.9 g.l^−1^. The fatty acid profile of the cells did not change significantly upon entry into the nitrogen limitation phase (Figure 1C). Lipid content began to change significantly after *ca.* 17.5 h. Lipid accumulation was characterized principally by an increase in C18:1n-9 content, with a mean specific accumulation rate of 0.006 g.g_CDW_^−1^.h^−1^, and by a smaller increase in C18:2n-6,9 and C16:0 content (0.002 g.g_CDW_^−1^.h^−1^). Although not being under optimal lipid accumulation conditions, carbon overflow was avoided since citrate production was very low [8].

### Identification of global transcriptomic responses during the fed-batch process

**Figure 2:**
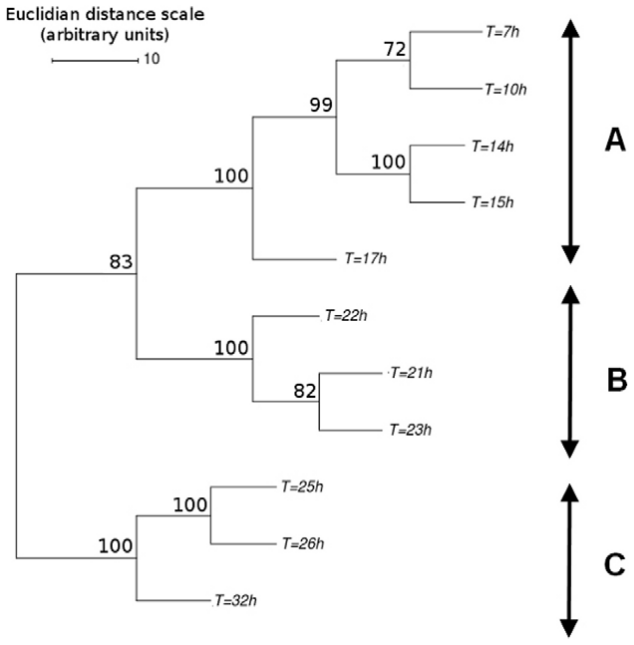
Hierarchical clustering of the global transcriptomic profiles obtained during the fed-batch culture. Bootstrap values were calculated from a 100 tree replicates. Three metabolic phases can be distinguished at the transcriptomic level: (A) biomass production, (B) early lipid accumulation, and (C) late lipid accumulation. Euclidian distance scale, as calculated by MeV software, is given in arbitrary units.

Hierarchical clustering analysis was carried out on the transcriptomic profiles of the samples, based on average linkage, Euclidean distance and 100 bootstrap replicates. The resulting dendrogram (Figure 2) highlights a sequential change in the global transcriptomic response. Different metabolic steps can thus be distinguished, mostly resembling those observed at the macroscopic scale, but with a few important differences. A first group of samples (Figure 2, group A) correspond to the growth phase, as identified in macrokinetic analyses. However, despite the switch to nitrogen-limiting conditions at *ca.* 15 h, the global transcriptomic response of these samples does not change until much later, with no major changes detected until T = 17 h. The transition phase is probably a transient state, highlighting a progressive transcriptomic response that will affect later stages of the fed batch culture. Finally, the lipid accumulation phase, as described in the macrokinetic approach, could be subdivided into two steps on the basis of transcriptomic analysis: an early phase (from T = 21 h to T = 23 h), and a late phase (from T = 25 h to T = 32 h). This distinction provides the first indication of a dual transcriptomic response to nitrogen-limiting conditions, which will be discussed further below.

### Differential expression during the transition to lipid accumulation

**Figure 3:**
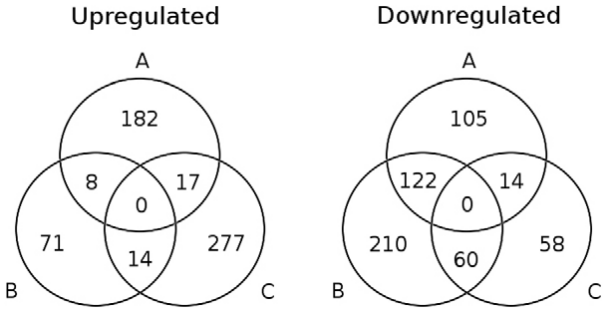
Distribution of genes identified as differentially expressed during the transition to lipid accumulation. Three metabolic states are distinguished during fed-batch : (A) biomass production, (B) early lipid accumulation, and (C) late lipid accumulation.

SAM tests were performed between each of the three transcriptomic response groups identified above. 569 different genes were identified as significantly over- or under-expressed in at least two of the three transcriptomic subsets (Table S2). Pairwise comparisons of the identified subsets provided a global overview of the transcriptomic response (Figure 3). We found that 207 genes were overexpressed during the biomass production phase, whereas 93 genes were significantly upregulated during the early accumulation phase and 308 genes were significantly upregulated during the late accumulation phase. Only a limited number of these genes (*ca.* 7%) appeared to be upregulated during two different phases, highlighting a time-related specificity of the upregulation response. Most of the genes upregulated after the switch to nitrogen-limiting conditions (*i.e.* genes detected as upregulated during either phase B and/or C) were expressed during the late accumulation phase (*ca.* 80%). Downregulation was observed for 241 genes during biomass production, 392 genes during the early accumulation phase and 132 genes during the late accumulation phase. The downregulation response appeared to be a slower, transient phenomenon, with large numbers of genes downregulated in consecutive phases. Most repressed genes (*ca.* 69%) are detected during the early lipid accumulation phase, highlighting the decrease in metabolic activity constituting the primary response to nitrogen starvation.

The genes identified as specifically expressed in one of the three transcriptomic phases were further classified into 20 functional categories, based on GO Terms (Table 1). Many metabolic processes were found to be involved in the various phases, but some functional categories were particularly frequently represented. In particular, genes involved in the cell cycle and in cellular component biogenesis and organization were numerous. A large proportion of these genes displayed downregulation after the imposition of nitrogen limitation, reflecting the slower growth observed at the start of the accumulation phase. Genes associated with translation followed a similar pattern. Other major categories identified were linked to the stress response, nucleic acid metabolism and transport. Lipid metabolism accounted for only a small proportion of genes, most of which were overexpressed during the late accumulation phase.

**Table 1:** Functional classification of the upregulated/downregulated genes identified during the transition to lipid accumulation.

	Number of genes up/downregulated during each transcriptomic phase		
Functional category	A	B	C
Amino acid metabolism	11/6	4/12	15/3
Carbohydrate metabolism	8/4	4/9	10/4
Catabolism	0/5	3/2	6/1
Cell cycle	12/10	4/17	34/3
Cellular aromatic compound metabolism	7/1	2/5	2/0
Cellular component biogenesis	34/4	1/25	14/3
Cellular component organization	52/17	11/51	64/12
Cellular metabolism	16/4	7/15	14/7
Lipid metabolism	2/3	1/12	17/0
Nucleic acids metabolism	48/14	5/39	46/8
Protein metabolism	1/1	0/1	5/0
Protein modification	13/8	4/16	19/4
Response to stimulus	20/12	10/24	36/5
Signalling	8/1	0/9	10/1
Transcription	7/7	3/7	20/3
Translation	74/2	1/52	10/1
Transport	29/22	14/28	42/8
Transposition	0/0	0/0	1/0
Vitamin metabolism	2/0	1/1	2/2
Unknown	37/45	30/65	119/27

(A) biomass production, (B) early lipid accumulation, and (C) late lipid accumulation. Functional categories were determined based on GO Terms defined during the annotation of *Y. lipolytica*, and by comparisons with homologous genes in *S. cerevisiae*.

**Figure 4:**
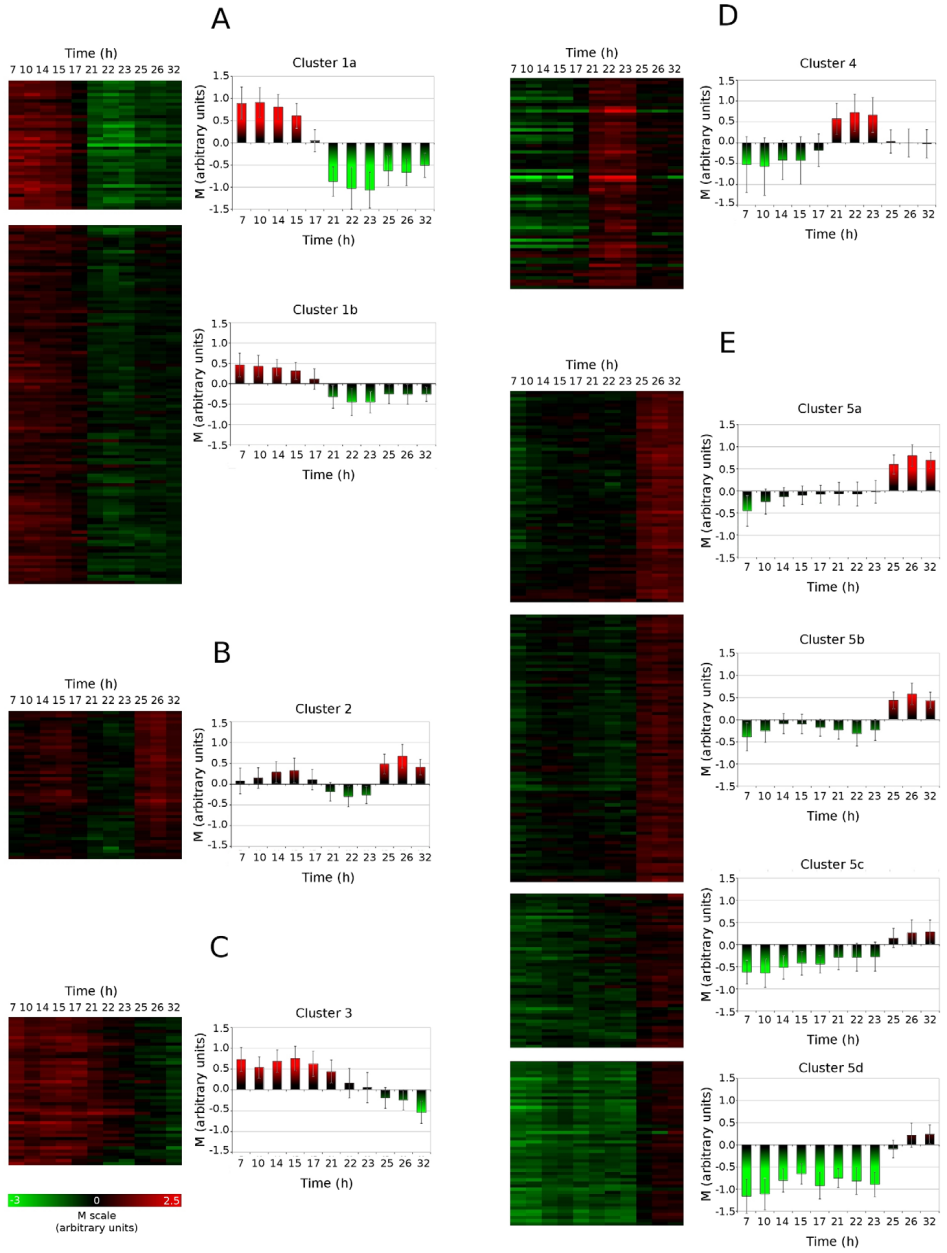
K-Mean clustering of the differentially expressed genes identified during the transition to lipid accumulation. The 569 genes (detailed in Table S2) were resolved into 9 clusters, which could further be classified into 5 response profiles: (A) downregulation upon nitrogen limitation, (B) transient repression, followed by late overexpression, (C) downregulation during late accumulation phase, (D) upregulation upon nitrogen limitation, and (E) upregulation during late accumulation phase. Mean expression values and error bars were calculated, based on the expression profiles of the genes identified in each clusters.

Additional classification by K-means clustering was performed to obtain further insight into the transcriptomic response (particularly as concerns the chain of metabolic events preceding and following nitrogen limitation). The transcriptomic profiles of the 569 differentially expressed genes were resolved into nine clusters, based on mean expression profiles (Figure 4). Some clusters could be further regrouped into similar response patterns, but with different intensities. Clusters 1 to 3 contained 249 genes repressed at one point during the course of the fed-batch process. Clusters 1a and 1b contained 43 and 112 genes, respectively, that were strongly repressed upon entry into the nitrogen limitation phase. Cluster 2 corresponds to a transient repression phenomenon and includes 47 genes downregulated in the early accumulation phase but recovering significant levels of expression in the late stages of lipid production. By contrast, cluster 3 included 47 genes that were strongly downregulated during the late accumulation phase. In parallel, clusters 4 and 5 contained 320 genes overexpressed at one point during the lipid accumulation phase. Cluster 4, in particular, may be considered to correspond to an immediate response to nitrogen limitation, whereas clusters 5a, 5b 5c and 5d contain mostly genes upregulated during the late accumulation phase. The major genes identified in these clusters are presented in Tables 2, 3 and S3, and their role in the chain of metabolic events is discussed below.

**Table 2:** Genes involved in energy and lipid metabolism, identified as differentially expressed during the transition to lipid accumulation.

				Fold Change (arbitrary units)		
Gene Labels	Gene Names	Description	Cluster	B/A	C/B	C/A
***Glyoxylate bypass***						
YALI0C16885g	*ICL*	Isocitrate lyase	1b	0.53	1.09	0.57
YALI0D19140g	*MLS1*	Malate synthase 1, glyoxysomal	4	2.09	0.29	0.61
***TCA cycle***						
YALI0D16753g	*MDH1*	Malate dehydrogenase, mitochondrial	3	0.67	0.85	0.57
YALI0D09361g	*ACO1*	Aconitase	5a	1.03	1.58	1.64
***Cellular energy homeostasis***						
YALI0B00704g	*ADK*	Adenylate kinase	5b	0.83	1.55	1.29
YALI0F26521g	*ADK2*	Adenylate kinase	4	1.2	0.64	0.77
***Oxidative phosphorylation***						
YALI0A20680g	*n.a.*	NADH dehydrogenase	2	0.76	1.58	1.21
YALI0E23089g	*n.a.*	NADH dehydrogenase	3	0.86	0.65	0.56
YALI0A02651g	*n.a.*	NADH dehydrogenase	5c	1.15	1.75	2
YALI0E08617g	*VMA13*	Vacuolar H+ ATPase, H subunit	3	1.16	0.54	0.62
YALI0F09405g	*PPA1/VMA16*	Vacuolar H+ ATPase, c” subunit	5b	1.1	1.86	2.05
YALI0B03982g	*ATP2*	ATP synthase, beta chain	3	0.68	0.77	0.52
***CoenzymeA synthesis***						
YALI0F09625g	*DPCK*	Dephospho-CoA kinase	4	1.71	0.89	1.52
***Pentose phosphate pathway***						
YALI0B00836g	*PRS5*	Ribose-phosphate pyrophosphokinase	1b	0.61	1.26	0.76
YALI0B15598g	*GND1*	6-phosphogluconate dehydrogenase	1b	0.76	0.82	0.62
YALI0F07711g	*PGI1*	Glucose-6-phosphate isomerase	3	1.35	0.59	0.8
***Glycolysis/gluconeogenesis***						
YALI0C06369g	*GAPDH*	Glyceraldehyde-3-P dehydrogenase	3	0.93	0.64	0.59
YALI0F07711g	*PGI1*	Glucose-6-phosphate isomerase	3	1.35	0.59	0.8
YALI0B02728g	*PGAM1*	Phosphoglycerate mutase	1b	0.66	0.93	0.61
YALI0E00264g	*ALD4*	Aldehyde dehydrogenase	5c	0.74	1.9	1.4
YALI0E17787g	*n.a.*	Alcohol dehydrogenase 2	1b	0.49	1.88	0.92
***Lipid metabolism***						
YALI0C05951g	*FAD1*	Delta-9 fatty acid desaturase	5c	0.87	1.65	1.44
YALI0F06578g	*DGA2*	Diacylglycerol acyltransferase	5a	1.16	1.89	2.2
YALI0D05995g	*SAC1*	Inositol/phosphatidylinositol phosphatase	5a	0.93	1.96	1.82
YALI0E11561g	*LIP15*	Lipase	5b	0.73	2.13	1.54
YALI0C03003g	*n.a.*	Peroxisomal 2,4-dienoyl-CoA reductase	5b	0.9	1.68	1.51
YALI0E14322g	*n.a.*	Peroxisomal 2,4-dienoyl-CoA reductase	5b	1.14	1.54	1.76
YALI0F23793g	*FALDH1*	Fatty aldehyde dehydrogenase	5b	1.13	1.45	1.65

Genes were identified as differentially expressed in cases of significant detection, with a false discovery rate lower than 1×10−5 and an absolute fold change between two groups of more than 1.5. Gene labels and descriptions were attributed according to the Genolevure database (http://www.genolevures.org/). Gene names were given, whenever possible, according to the identification of homologous genes in *S. cerevisiae* (n.a. : not available).

**Table 3:** Regulators and transcription factors identified as differentially expressed during the transition to lipid accumulation.

				Fold Change (arbitrary units)		
Gene Labels	Gene Names	Description	Cluster	B/A	C/B	C/A
YALI0F30173g	*TFB2*	TFIIH transcription factor subunit	1b	0.61	0.9	0.54
YALI0F03630g	*n.a.*	Putative zinc finger transcription factor, Zn(2)-Cys(6) family	3	0.92	0.67	0.62
YALI0A14542g	*TUP1*	General repressor of transcription	5a	1.1	1.78	1.95
YALI0D02673g	*PTR3*	Component of the SPS amino-acid sensor complex	5a	1.23	1.28	1.58
YALI0D09647g	*n.a.*	Putative arginine metabolism regulation protein	5a	1.04	1.47	1.52
YALI0F02783g	*NPR2*	Putative nitrogen permease regulator NPR2-like	5a	1.1	1.54	1.7
YALI0F17424g	*Hap1*	Putative zinc finger transcription factor, HAP1-like activator	5a	1.19	1.75	2.07
YALI0D20482g	*n.a.*	Putative nitrogen regulatory protein, AREA-like	4	1.68	0.62	1.04
YALI0E18986g	*UBI4*	Ubiquitin	4	2.87	0.67	1.92
YALI0D13046g	*Otu1*	Putative zinc finger transcription factor, OTU1-like	5c	1.53	0.99	1.51
YALI0C19151g	*Cat8*	Putative zinc finger transcription factor, CAT8-like	5d	1.16	1.71	1.99
YALI0A10637g	*Hal9*	Putative zinc finger transcription factor, HAL9-like	4	1.51	0.95	1.44
YALI0E29909g	*PDR6*	Pleiotropic drug resistance regulatory protein	5b	0.74	1.94	1.43
YALI0B12716g	*HAC1*	Basic leucine zipper transcription factor, involved in the unfolded protein response	5b	1.09	2.05	2.24

Genes were identified as differentially expressed in cases of significant detection, with a false discovery rate lower than 1×10−5 and an absolute fold change between two groups of more than 1.5. Gene labels and descriptions were attributed according to the Genolevure database (http://www.genolevures.org/). Gene names were given, whenever possible, according to the identification of homologous genes in *S. cerevisiae* (n.a. : not available).

## Discussion

Lipid accumulation phenotypes have been known for a long time, but the biochemical differences between oleaginous and non oleaginous organisms remain to be elucidated. Despite the development of high-throughput sequencing, transcriptomic and proteomic methods, together with economic and industrial interest in oil production, “-omics” information for oleaginous yeasts remains scarce. Only a limited number of proteomics studies in recent years have focused on the mechanisms of lipid accumulation [20], [21], [38].

*Y. lipolytica* has been shown to accumulate up to 36% of its dry weight as lipid [7]. However, recent studies on strain ACA-DC 50109 growing on glucose as the sole carbon source highlighted problems achieving lipid accumulation rates of more than 20%, despite strong nitrogen limitation and high C/N ratios [39]. Together with a preferential use of hydrophobic substrates, these results called into question the potential of *Y. lipolytica* for lipid production an accumulation *via* a *de novo* fatty acid biosynthesis pathway [20].

In this study, we used a controlled fed-batch set-up to analyze the behavior of *Y. lipolytica* during the transition from growth to lipid accumulation. Culture parameters were carefully modulated to control the induction of lipid accumulation. The calculated glucose and nitrogen flows implied that lipid production was suboptimal with respect to the maximum levels obtained in fed-batch mode [23], [40]. In particular, dual nitrogen and glucose limitation was used in later stages of fed-batch culture, to prevent excess citric acid production that might ultimately partly mask the transcriptomic response. Despite these suboptimal conditions, our results clearly demonstrate that *Y. lipolytica* can accumulate lipids synthesized *de novo*, using glucose as the sole carbon source.

### Transcriptomic response of an oleaginous yeast to nitrogen-limiting conditions

One of the primary consequences of nitrogen limitation is a decrease in cell proliferation. As already observed in the proteomes of other oleaginous yeast species, such as *L. starkeyi* and *R. toruloides* [20], [21], much of the transcriptomic response of *Y. lipolytica* reflects the decrease in growth rate upon nitrogen limitation. Clusters 1a and 1b contain a large number of genes related to cellular metabolism, cell growth and particularly protein synthesis, including 61 genes encoding ribosomal subunits, two translation initiation factors, and seven translation elongation factors (Table S2).

Meanwhile, the assimilated glucose is redirected towards the citric acid cycle to provide lipid biosynthesis, initiated by the fatty acid synthase (FAS), from acetyl-CoA, malonyl-CoA and NADPH [4], [5]. Acetyl-CoA is cleaved from citrate within the cytosol by ATP:citrate lyase (ACL). This protein has been identified as a key enzyme of lipid accumulation in oleaginous organisms, even though some non-oleaginous species exhibit ACL activity [7]. ACL is considered the main provider of Acetyl-CoA for both FAS, and Acetyl-CoA Carboxylase (ACC) that will further provide the FAS with malonyl-CoA [7]. On the other hand, malic enzyme (ME) is considered to be the supplier of NADPH in most oleaginous species. All four enzymes have been found in *Y. lipolytica*, but neither displays a significant change in transcription level in response to nitrogen limitation. This could be easily interpreted for the ME, as in *Y. lipolytica*, contrary to most oleaginous yeasts, only a single mitochondrial form of the enzyme is predicted to exist. Therefore it should be unable to provide the NADPH to the cytosolic FAS. Meanwhile, several enzymatic studies in oleaginous yeasts have shown that ACL activity increases during lipid accumulation [41]. Thus, if similar increases in ACL levels occur in *Y. lipolytica*, they are presumably regulated posttranslationally. As for ACC, transcriptomic data were below our quality threshold, which led us to the incapacity to draw any conclusion for now regarding its expression under nitrogen limiting conditions.

While the main genes governing fatty acid synthesis do not seem to be directly controlled at the transcriptomic level, lipid accumulation could also be a passive consequence of the rerouting of carbon fluxes leading to the production of Acetyl-CoA. Acetyl-CoA production may depend on a sequence of biochemical events, beginning with an increase in AMP deaminase activity shortly after nitrogen limitation [42]. Transcriptomic data tend to highlight a slight increase in expression of the related gene *AMD1* (data not shown), but this increase is not statistically significant, suggesting that the sharp increase in AMP deaminase activity, like the one reported for AMD in *Rhodosporidium toruloides* [7], may also be induced by posttranslational modifications rather than through transcriptional control. Several genes encoding proteins involved in protein modification displayed significant overexpression during the fed-batch process (Table 1, and Table S2) and are therefore potential targets of choice for future investigations of the impact of posttranslational modification on the metabolism of oleaginous yeasts.

Another key regulation point is the inhibition of the AMP-dependent isocitrate dehydrogenase (IDH) in the TCA cycle [4], [7]. In our study, no significant change in *IDH* expression was detected during lipid accumulation either. However, Morgunov and colleagues [43] isolated IDH from *Y. lipolytica* subjected to nitrogen starvation and *in vitro* enzymatic activity tests showed that the enzyme was still present and functional under nitrogen limiting conditions. The decrease in IDH activity could therefore be controlled by cellular AMP content rather than transcriptionally. In parallel, the gene YALI0C16885g, encoding isocitrate lyase (ICL), seems to be strongly and immediately repressed by nitrogen limitation (Table 2). ICL is a peroxisomal enzyme involved in the glyoxylate pathway, in which it converts isocitrate into glyoxylate and succinate [44]. A strong decrease in ICL activity has also been reported in several oleaginous yeasts under nitrogen-limiting conditions [41]. With the presumably low levels of IDH activity [7], and the repression of *ICL* transcription, isocitrate cannot be metabolized through the TCA or glyoxylate cycles. However, the accumulating isocitrate can be rerouted within the TCA to produce citrate, through the action of aconitase (YALI0D09361g). Interestingly, aconitase would be expected to be equally active under conditions of carbon and nitrogen limitation [7], but its gene was significantly more strongly expressed during the late phase of lipid accumulation, favouring citrate accumulation (Table 2). When put together, our transcriptomic results tend to confirm the re-routing of the TCA towards citrate accumulation. Citrate can be further provided to ACL, *via* the action of the Citrate/Malate Translocase (CMT) [7]. The gene encoding this particular transport system has not been identified in the genome of *Yarrowia* so far. Transcriptomic results highlighted several genes encoding transport systems, some of which are over-expressed during lipid accumulation (Table S2). However, none of them appear at this stage as a potential candidate for a mitochondrial translocase system.

While citrate seems to be a key metabolite for lipid accumulation [7], ACL also requires both CoA and ATP for the synthesis of acetyl-CoA. It is interesting to notice that several genes involved in the metabolism of CoA and ATP display significant changes in their expression during the lipid accumulation process. Notably, the gene encoding dephospho-CoA kinase (DPCK, YALI0F09625g), the final enzyme in the CoA biosynthesis pathway, was significantly overexpressed during the late phase of lipid accumulation (Table 2). DPCK plays an important role in regulating CoA biosynthesis [45]. The overexpression of this gene may be an indicator of an increase in the CoA pool during lipid accumulation. As for ATP, its intracellular concentration was studied in various oleaginous yeasts in which no significant changes were highlighted during lipid accumulation [7]. However, ATP, ADP and AMP levels have been reported to fluctuate in *Y. lipolytica* under nitrogen-limiting conditions [43]. In our study, several genes involved in ATP metabolism have been identified as displaying differential expression in response to nitrogen limitation in *Y. lipolytica*, such as (i) *ADK1* and *ADK2*, encoding two adenylate kinases involved in cellular energy homeostasis [46], (ii) genes involved in oxidative phosphorylation, (iii) the *ATP2* gene, encoding the mitochondrial ATP synthase beta chain [47], (iii) genes coding for ATPases subunits. All these observations suggest that the adenine nucleotide pool in *Y. lipolytica* during the transition to lipid accumulation may evolve more dynamically than what has been previously reported in other oleaginous yeasts, such as *L. starkeyi* [7]. As CoA and ATP are essential metabolites in acetyl CoA synthesis, they could ultimately affect lipid synthesis indirectly, *via* their mobilization and/or their availability for ACL.

Subsequent steps in lipid synthesis, such as elongation and desaturation, appear more likely to be controlled at the transcriptomic level. In particular, the gene encoding the delta-9 fatty acid desaturase [7] was overexpressed during lipid accumulation (Table 2). The expression of this gene was directly correlated with the accumulation of C18:1, as shown by the lipid profile of the fed batch (Figure 1c). Moreover, two genes related to lipid storage are significantly expressed in the late stage of lipid accumulation: (i) *SAC1*, encoding an inositol/phosphatidylinositol phosphatase previously shown to be a component of lipid particles in *Y. lipolytica* [38], and (ii) *DGA2*, encoding a diacylglycerol acyltransferase. More specifically, *DGA2* has been recently found to encode a member of the type 1 acyl-CoA:diacylglycerol acyltransferase family (DGAT1), which has not previously been identified in yeasts, but is commonly found in mammals and plants [48]. The enzyme Dga2p has been highlighted as a major contributor to TAG synthesis in *Y. lipolytica*, *via* an acyl-CoA-dependent mechanism. Furthermore, expression of this enzyme not only contributes to TAG synthesis, but also affects the size and morphology of lipid bodies. The regulation of these two genes implies an intense lipid storage activity, although the expression rates of the three remaining acyltransferases (*i.e. DGA1*, *LRO1* and *ARE1*) were not significantly altered during culture. Interestingly, a lipase encoding gene, *LIP15*, is also over-expressed during lipid accumulation, which could suggest that lipid turnover proceeds in parallel with lipid accumulation. *Y. lipolytica* possesses many genes coding for lipases, among which (i) two genes coding for intracellular TAG lipases homologous to *S. cerevisiae*, namely *TGL3* and *TGL4* [18], and (ii) 16 paralogs of genes coding for lipase, regrouped to form the *LIP* family [49]. However, little information is known about the role and specificity of each member of this lipolytic arsenal. Only three isoenzymes (Lip2p, Lip7p and Lip8p) have been partly characterized so far [49]. Hence, the exact function of the protein encoded by the *LIP15* gene has yet to be fully resolved. It could thus represent a target of choice for future analyses, especially since some genes involved in TAG degradation such as *TGL5* have yet to be discovered in *Yarrowia* [18]. The enhancement of lipid storage capacity combined with the repression of lipid turnover have already proven to be effective strategies to improve lipid accumulation in *Y. lipolytica* [18], [50]. Genes highlighted in our study, such as *DGA2*, or *LIP15* could be potential targets for genetic manipulation in order to alter the lipid accumulation capacity of *Y. lipolytica*.

Finally, several glycolysis genes were repressed during lipid accumulation (Table 2). Previous studies have emphasized the negative feedback control of phosphofructokinase (PFK) and pyruvate kinase (PK), the presumed principal regulators of glucose uptake, by citrate accumulation [7]. Our transcriptomic study also highlights the transcriptional regulation of genes involved in glycolysis, indicating a global gene response to the carbon overflow induced by nitrogen limitation [8]. It is therefore probable that repression of glycolysis eventually limits lipid accumulation on the long term. Improvements in our understanding of the regulation mechanisms involved in glucose uptake could lead to the identification of alternative targets for the production of over-accumulating strains.

### Adaptation of *Y. lipolytica* to nitrogen limitation

Yeasts and fungi are able to utilize diverse nitrogen sources, including ammonium, amino acids, urea, nitrogen bases and purine derivatives as nutriment for growth [51], [52]. During culture, specific permeases are synthesized, permitting the incorporation of the nitrogen containing substrate [53]. These nitrogen sources may either be used directly in biosynthetic pathways or catabolized to generate ammonium, glutamate, and glutamine. In the fed-batch process used in this study, nitrogen limitation is induced by the temporary cessation of ammonium supplementation. It is therefore unsurprising that much of the transcriptomic data collected here reflects upon the transition of the cell metabolism in search for an alternative nitrogen source. The genes differentially expressed during this nitrogen-induced transition are presented in Table S3.

The above phenomena, however, are regulated by complex mechanisms involving several interacting and competing regulatory systems, some of which have activities beyond the scope of nitrogen metabolism [51], [52]. Specific transcriptional controls affecting single biosynthetic pathways (*e.g.* arginine, branched-chain amino acids, lysine, methionine) have been described [54]–[57], but are not yet entirely understood in *Y. lipolytica*. One gene encoding a protein involved in the regulation of arginine metabolism (YALI0D09647g) was found to be expressed in the late stage of lipid accumulation (Table 3). However, the repression of genes involved in aromatic amino-acid biosynthesis may also reflect the general control of amino-acid biosynthesis (GAAC) [58]. Regulation of the GAAC in *S. cerevisiae* is reportedly mediated by the transcription factor Gcn4 [52], [58], for which an homolog has been identified in *Y. lipolytica* (YALI0E27742g), but no significant expression could detected under nitrogen limitation.

A large proportion of the genes identified above are subject to nitrogen catabolite repression (NCR) [51], [52]. A GATA-like transcription factor (YALI0D20482g) appears to become particularly active upon nitrogen limitation (Table 3). Similar factors have been linked to NCR regulation in fungi and yeasts [51], [59], [60]. In *S. cerevisiae*, NCR involves the inhibition of two transcription factors of the GATA family: Gln3 and Gat1/Nil1 [61], [62]. These transcription factors recognize a 5′-GATA-3′ sequence located upstream from genes subject to NCR. Gln3 and Gat1/Nil1 have high levels of sequence similarity, but differ in their expression patterns. Gln3 is constitutively expressed in *S. cerevisiae* and is inhibited by Ure2 at high nitrogen levels [60]. By contrast, the expression of *Gat1/Nil1* is repressed under normal conditions by another GATA family transcription factor, DEH1/Gzf3 [65]. Under specific nitrogen conditions, a fourth GATA factor, Dal80, has also been shown to inhibit *Gat1* [60]–[62]. Three GATA transcription factor genes have been identified in *Y. lipolytica*: (i) one encoding a homolog of Gzf3 (YALI0C22682g) and (ii) two encoding Gat1/Gln3-like factors (YALI0D20482g, YALI0F17886g). As in *S. cerevisiae*, these two Gat1/Gln3-like factors identified in *Y. lipolytica* display high levels of sequence similarity. However, only YALI0D20482g has been shown to be differentially expressed under nitrogen-limiting conditions. Therefore, we would expect this gene to encode a Gat1-like transcription factor, whereas YALI0F17886g encodes a constitutively produced Gln3-like protein.

Finally, additional genes linked to the regulation of nitrogen metabolism were identified from the transcriptomic data (Table 3). One such gene, *Ptr3*, encodes a subunit of the Ssy1-Ptr3-Ssy5 (SPS) sensor complex. This complex is involved in ammonium source detection and has been linked to the control of various permease-encoding genes [63], [64]. Another, the *NPR2* gene, belongs to a family of regulators involved in the posttranslational control of nitrogen permease [65].

### Regulation

The general mechanisms regulating lipid accumulation in oleaginous organisms have yet to be completely elucidated, despite the enormous potential they represent. Through transcriptomic analyses of *Y. lipolytica*, we have highlighted the action of several transcription factors and regulatory proteins, some of which had already been identified as potentially important (Table 3). In particular, YALI0F30173g, which encodes a subunit of the transcription factor TFIIH, appears to be strongly repressed by nitrogen limitation. TFIIH is a known general transcription factor involved in the RNA polymerase II preinitation complex and in nucleotide excision repair [66]. Similarly, YALI0A14542g encodes a TUP1-like general repressor of transcription activated within hours of nitrogen limitation. The regulation of these two factors may be linked to the repression of transcription and protein synthesis observed following nitrogen limitation.

Five genes encoding regulatory proteins of the zinc cluster family were identified as differentially expressed upon nitrogen limitation. Zinc cluster proteins (ZCP) are named after a family of zinc-containing structural motifs generally associated with DNA, RNA or protein-binding properties [67]. As such, ZCP proteins include diverse transcriptional factors involved in the regulation of various metabolic processes [67], [68]. One of these factors, encoded by YALI0F03630g, was also found to be repressed, mostly at the late stage of lipid production. However, the sequence of this gene displays little similarity to those of yeast transcription factors of unknown function.

Four of these five genes encode transcription factors of the zinc cluster family. The four ZCP-like transcription factors identified are putative homologs of *Hap1*, *Otu1*, *Cat8* and *Hal9*. The *Hap1* gene encodes a yeast heme activator protein that promotes the transcription of many genes in response to heme and oxygen [69]. Otu1 has hydrolase activity responsible for removing conjugated ubiquitin from proteins, thus potentially playing an important role in regulating protein turnover by preventing degradation [70]. It has been shown to participate in the regulation of ubiquitin, which was also significantly expressed (Table 3). *Cat8* encodes a known activator of gluconeogenic enzymes [71]. Finally, *Hal9* encodes a putative transcription factor involved in halotolerance [72].

Finally, two additional transcription factors, involved in pleiotropic drug resistance and unfolded protein response, were detected in cluster 5b. Interestingly, these two metabolic processes were previously identified as responding to changes in nitrogen sources in *S. cerevisiae*, despite a lack of direct involvement in nitrogen metabolism [52]. This finding confirms the observations made on the baker's yeast transcriptome, but also highlights the complexity and interconnectivity of regulatory mechanisms in yeast.

### Concluding remarks

In conclusion, this study highlights genes potentially involved in the oleaginous characters of the cell at the transcriptomic level. The expression of genes encoding several key enzymes, such as ACL, ME, AMD and IDH, does not seem to be regulated at the transcriptional level. However, a complex cascade of transcriptional events may lead to the increase of the various substrate and cofactor pools necessary for the synthesis of both acetyl-CoA and NADPH. These findings are consistent with the hypothesis that lipid accumulation in oleaginous yeasts is a consequence of the rerouting of carbon fluxes upon nitrogen limitation, rather than specific and/or enhanced lipid metabolism activity.

## Supporting Information

Table S1 **Growth conditions, composition of the fed-batch medium, salts and vitamins stock solutions.**
(DOC)

Table S2 **Description of the 569 genes identified as differentially expressed during the transition to lipid accumulation.**
(XLS)

Table S3 **Genes involved in nitrogen-metabolism identified as differentially expressed during the transition to lipid accumulation.**
(DOC)
