# Supplementary material for: Transcriptomic Analyses during the Transition from Biomass Production to Lipid Accumulation in the Oleaginous Yeast Yarrowia lipolytica
Source: PLoS One. 2011 Nov 22;6(11):e27966. doi: 10.1371/journal.pone.0027966 (PMC3222671; doi:10.1371/journal.pone.0027966)
Supplement: Table S1 — Growth conditions, composition of the fed-batch medium, salts and vitamins stock solutions. (DOC) [file pone.0027966.s001.doc]

**Supplementary Table S1 : Growth conditions, composition of the fed-batch medium, salts and vitamins stock solutions.** Axenic cultures were carried out in rich medium (lysogeny broth, LB) containing 10 g.l^-1^ glucose. Cells were cultured in flasks, with shaking, and harvested before the stationary growth phase. Sterile glycerol was added (30 % v/v) to 1 ml aliquots in sterile vials, which were frozen and stored at – 80 °C. These frozen stock cultures were used to inoculate the fed batch culture. Inoculum was prepared as follows: a preliminary batch culture was performed in a 100 ml baffled Erlenmeyer flask containing 8 ml of LB (30 °C, 16 h, shaking at 100 rpm). The culture medium was transferred to a 250 ml baffled Erlenmeyer flask containing 72 ml of mineral medium, with 0.1% (v/v) vitamin solution and 10 g.l^–1^ glucose, and incubated for 12 h at 30 °C. The composition of preculture and culture media and solutions (see table below) were defined as described by Egli and Fiechter [1]. The resulting 80 ml culture was used to inoculate a 5 l Erlenmeyer flask containing 720 ml of mineral medium with 0.1% (v/v) vitamin solution, which was then incubated at 30°C for 12 h. This culture was used to inoculate 7.2 l of mineral medium directly in the bioreactor. The bioreactor was supplied with a constant ratio of mineral medium to substrate of 1:10. The composition of the mineral medium input is described in supplementary Table S1. Vitamin solution was added during biomass growth, with 10 ml of vitamin solution added for every 10 g_CDW_.l^-1^ of newly formed biomass [2]. Vitamins were at least 99.9% pure and were obtained from Sigma-Aldrich (Sigma-Aldrich Chimie SARL, France). Glucose was provided by Roquette (France). All other chemicals (*e.g.* mineral medium, solvent, mobile phases) were at least 99% pure and were purchased from VWR (France).

1. Egli T, Fiechter A (1981) Theoretical Analysis of Media Used in the Growth of Yeasts on Methanol. Microbiol 123: 365-369.

2. Alfenore S, Molina-Jouve C, Guillouet SE, Uribelarrea J-L, Goma G, Benbadis L (2002) Improving ethanol production and viability of *Saccharomyces cerevisiae* by a vitamin feeding strategy during fed-batch process. Appl Microbiol Biotechnol 60: 67-72.

| **Solution** | **Component** | **Concentration (g.L-1)** |
| --- | --- | --- |
| Preculture and Starting medium | K2HPO4 | 3.0 |
|  | NaH2PO4 ; H2O | 3.0 |
|  | (NH4)2SO4 | 3.0 |
|  | MgSO4·; 7H2O | 1.0 |
|  | ZnSO4·; 7H2O | 0.0 |
|  | FeSO4·; 7H2O | 0.016 |
|  | MnSO4·; H2O | 0.0038 |
|  | CoCl2·; 6H2O | 0.0005 |
|  | CuSO4·; 5H2O | 0.0009 |
|  | Na2MoO4·; 2H2O | 0 |
|  | CaCl2·; 2H2O | 0.023 |
|  | H3BO3 | 0.003 |
| Mineral solution | KCl | 20.0 |
|  | NaCl | 20.0 |
|  | MgSO4 ;·7H2O | 27.0 |
|  | ZnSO4·; 7H2O | 7.71 |
|  | MnSO4·; H2O | 0.47 |
|  | CoCl2·; 6H2O | 0.3 |
|  | CuSO4·; 5H2O | 0.6 |
|  | Na2MoSO4·; 2H2O | 0.094 |
|  | CaCl2·; 2H2O | 6.4 |
|  | FeSO4·; 7H2O | 3.97 |
|  | H3BO3 | 0.3 |
|  | H3PO4 | 125.0 |
| Vitamin solution | d-Biotine | 0.1 |
|  | Panthotenate | 1.0 |
|  | Nicotinic acid | 1.0 |
|  | Hydrochloride thiamine | 1.0 |
|  | P-amino-benzoic acid | 0.2 |
|  | Hydrochlorid pyridoxol | 1.0 |
|  | Myoinositol | 25.0 |
