# Supplementary material for: Transcriptomic Analyses during the Transition from Biomass Production to Lipid Accumulation in the Oleaginous Yeast Yarrowia lipolytica
Source: PLoS One. 2011 Nov 22;6(11):e27966. doi: 10.1371/journal.pone.0027966 (PMC3222671; doi:10.1371/journal.pone.0027966)
Supplement: Table S3 — Genes involved in nitrogen-metabolism identified as differentially expressed during the transition to lipid accumulation. (DOC) [file pone.0027966.s003.doc]

**Supplementary Table S3: Genes involved in nitrogen-metabolism identified as differentially expressed during the transition to lipid accumulation.**

|  |  |  |  | **Fold Change (arbitrary units)** | | |
| --- | --- | --- | --- | --- | --- | --- |
| **Gene Labels** | **Gene Names** | **Description** | **Cluster** | **B/A** | **C/B** | **C/A** |
| ***Amino-acid metabolism and derivatives*** | | | | | | |
| YALI0B15304g | *ADE4* | Amidophosphoribosyltransferase | 1b | 0.61 | 1.18 | 0.73 |
| YALI0D24409g | *ADE17* | Phosphoribosylaminoimidazolecarboxamide formyltransferase | 1b | 0.61 | 1.09 | 0.67 |
| YALI0D14894g | *ARG3* | Ornithine carbamoyltransferase | 1b | 0.65 | 1.15 | 0.75 |
| YALI0B20020g | *ARO3* | 3-deoxy-7-phosphoheptulonate synthase | 1b | 0.61 | 1.09 | 0.66 |
| YALI0E24607g | *CDC60* | Leucyl-tRNA synthetase | 1b | 0.94 | 0.63 | 0.59 |
| YALI0B16104g | *GUA1* | GMP synthase | 1b | 0.53 | 1.3 | 0.69 |
| YALI0D11704g | *HOM3* | Aspartate kinase | 1b | 0.58 | 1.39 | 0.8 |
| YALI0D03135g | *ILV5* | Ketol-acid reductoisomerase | 1a | 0.43 | 1.05 | 0.45 |
| YALI0F16291g | *KRS1* | Lysyl-tRNA synthetase | 1b | 0.66 | 1.05 | 0.69 |
| YALI0D10593g | *LYS12* | Homocitrate dehydrogenase | 3 | 0.45 | 1.2 | 0.54 |
| YALI0B08184g | *MET3* | ATP sulfurylase | 1a | 0.29 | 1.54 | 0.45 |
| YALI0E12683g | *MET6* | Methionine synthase | 3 | 0.95 | 0.47 | 0.45 |
| YALI0B17336g | *PHA2* | Prephenate dehydratase | 1b | 0.63 | 0.98 | 0.62 |
| YALI0E09493g | *URA2* | Aspartate carbamoyltransferase catalytic chain | 1b | 0.64 | 1.18 | 0.75 |
| YALI0B05368g | *n.a.* | CTP synthase | 1b | 0.51 | 1.28 | 0.66 |
| YALI0B23100g | *n.a.* | Dihydrodipicolinate synthase | 1b | 0.57 | 1.01 | 0.57 |
| YALI0B14509g | *n.a.* | SAM synthetase | 3 | 0.66 | 0.89 | 0.59 |
| YALI0D06215g | *n.a.* | Putative mitochondrial 3-hydroxyisobutyryl-CoA hydrolase | 4 | 1.71 | 0.65 | 1.11 |
| ***Proline catabolism*** | | | | | | |
| YALI0E17171g | *PUT1* | Proline oxidase | 5a | 1.12 | 1.61 | 1.8 |
| YALI0B09647g | *PUT2* | Pyrroline 5-carboxylate dehydrogenase | 5d | 0.95 | 1.95 | 1.85 |
| ***Purine metabolism*** | | | | | | |
| YALI0A06974g | *n.a.* | Xanthine oxidase | 4 | 8.94 | 0.14 | 1.22 |
| ***Protein catabolism*** | | | | | | |
| YALI0F31889g | *n.a.* | Alkaline extracellular protease | 4 | 1.75 | 0.78 | 1.36 |
| YALI0A09262g | *n.a.* | Alkaline extracellular protease | 5b | 1.01 | 1.73 | 1.74 |
| YALI0C20691g | *n.a.* | Alkaline extracellular protease | 5c | 0.97 | 1.61 | 1.56 |
| YALI0E28875g | *n.a.* | Alkaline extracellular protease | 5d | 1.15 | 2.7 | 3.12 |
| ***Glutamine synthesis*** | | | | | | |
| YALI0F17820g | *GDH3* | Glutamate dehydrogenase (NADP+) | 4 | 1.5 | 0.54 | 0.82 |
| YALI0D13024g | *GLN1* | Glutamine synthetase | 4 | 2.65 | 0.55 | 1.47 |
| ***Transport*** | | | | | | |
| YALI0F30063g | *AVT1* | Neutral amino-acid transporter | 5a | 1.03 | 2.17 | 2.24 |
| YALI0D05621g | *DAL4* | Allantoin permease | 4 | 1.84 | 0.71 | 1.31 |
| YALI0B16412g | *DAL5* | Allantoate permease | 5a | 1.12 | 1.46 | 1.63 |
| YALI0E07271g | *DUR1,2* | Urea transporter | 4 | 15.14 | 0.11 | 1.74 |
| YALI0B04202g | *DUR3* | Urea transporter | 4 | 1.6 | 0.56 | 0.9 |
| YALI0F30569g | *FCY2* | Purine-cytosine permease | 5a | 1.67 | 0.76 | 1.27 |
| YALI0E27203g | *MEP2* | Ammonium permease | 5c | 1.53 | 1.06 | 1.62 |
| YALI0D16137g | *MUP1* | Methionine permease | 5c | 1.08 | 1.44 | 1.56 |
| YALI0B01914g | *SEO1* | MFS transporter, allantoate subfamily | 4 | 4.64 | 0.62 | 2.88 |
| YALI0E27852g | *UAPC* | Putative purine permease | 4 | 1.96 | 0.6 | 1.18 |
| YALI0A19228g | *n.a.* | Putative ammonium transporter | 5c | 1.4 | 1.6 | 2.24 |
| YALI0B16522g | *n.a.* | General amino acid permease, GAP1-like | 5d | 0.94 | 1.67 | 1.57 |
| YALI0C09889g | *n.a.* | General amino acid permease, GAP1-like | 5b | 0.91 | 1.87 | 1.7 |
| YALI0C17237g | *n.a.* | General amino acid permease, GAP1-like | 4 | 3.97 | 0.74 | 2.93 |
| YALI0E10219g | *n.a.* | General amino acid permease, GAP1-like | 5c | 1.22 | 1.35 | 1.64 |
| YALI0D19646g | *n.a.* | Methionine permease | 4 | 1.51 | 0.98 | 1.48 |
| YALI0E35200g | *n.a.* | MFS transporter, allantoate subfamily, SEO1-like | 5d | 1.02 | 1.87 | 1.9 |
| ***Other*** | | | | | | |
| YALI0E22627g | *DCG1* | Nitrogen catabolite metabolism protein | 5a | 1.23 | 1.42 | 1.74 |
| YALI0C22792g | *AMO1* | Amine oxidase | 4 | 2.14 | 0.64 | 1.37 |
| YALI0C18315g | *AMO2* | Amine oxidase | 4 | 5.83 | 0.6 | 3.49 |
| YALI0B16500g | *PRB1* | Protease B, vacuolar | 4 | 3.42 | 0.79 | 2.71 |
| YALI0E00264g | *ALD4* | Aldehyde dehydrogenase | 5c | 0.74 | 1.9 | 1.4 |
| YALI0F09691g | *n.a.* | Conserved hypothetical protein, HRD799-like | 4 | 1.59 | 0.83 | 1.32 |
| YALI0F25333g | *CPS1* | Vacuolar carboxypeptidase | 5d | 0.91 | 2.45 | 2.23 |

Genes were identified as differentially expressed in cases of significant detection, with a false discovery rate lower than 1 x 10^-5^ and an absolute fold change between two groups of more than 1.5. Gene labels and descriptions were attributed according to the Genolevure database (http://www.genolevures.org/). Gene names were given, whenever possible, according to the identification of homologous genes in *S. cerevisiae* (n.a. : not available).
